# Thiophene and diaminobenzo- (1,2,5-thiadiazol)- based DAD-type near-infrared fluorescent probe for nitric oxide: A theoretical research

**DOI:** 10.3389/fchem.2022.990979

**Published:** 2023-01-09

**Authors:** X. Y. Lin, S. H. Sun, Y. T. Liu, Q. Q. Shi, J. J. Lv, Y. J. Peng

**Affiliations:** ^1^ College of Public Health, Jinzhou Medical University, Jinzhou, China; ^2^ College of Bio informational Engineering, Jinzhou Medical University, Jinzhou, China; ^3^ College of Physics, Nankai University, Tianjin, China

**Keywords:** fluorescent probe, nitric oxide, inflammatory bowel disease, density functional theory, electron transfer

## Abstract

A near-infrared fluorescent probe (LS-NO) for the real-time detection of nitric oxide (NO) in inflammatory bowel disease (IBD) was developed recently. The probe used oligoglycol morpholine-functionalized thiophene as strong electron donors and diaminobenzene (1,2,5-thiadiazole) as a weak electron acceptor and NO trapping group. It could detect exogenous and endogenous NO in the lysosomes of living cells with high sensitivity and specificity. To further understand the fluorescent mechanism and character of the probes LS-NO and LS-TZ (after the reaction of the probe LS-NO with NO), the electron transfer in the excitation and emitting process within the model molecules DAD-NO and DAD-TZ was analyzed in detail under the density functional theory. The calculation results indicated the transformation from diaminobenzene (1,2,5-thiadiazole) as a weak electron acceptor to triazolo-benzo-(1,2,5-thiadiazole) as a strong electron acceptor made LS-NO an effective “off–on” near-infrared NO fluorescent probe.

## 1 Introduction

Nitric oxide (NO) is a metastable free radical molecule which plays an important role in signal transduction and regulation in cardiovascular, immune, respiratory, gastrointestinal, and central nervous systems, and other physiological systems (Wang et al., 2002; Cosby et al., 2003). Recent studies have demonstrated that NO disorders are associated with a number of human diseases, including atherosclerosis, immune diseases, neurodegenerative diseases, cancer, and inflammatory bowel disease (Bogdan, 2001; Mel et al., 2011; Fmedsci, 2016). Moreover, increasing the NO concentration in the intestine is closely related to IBD (Kamalian et al., 2020). However, due to the lack of an effective method for the real-time detection of NO in the intestinal tract, the mechanism between NO and the pathogenesis of IBD remains unclear. Therefore, there is an urgent need to develop NO imaging probes with high sensitivity, high specificity, and high spatial and temporal resolution for the real-time detection of NO *in vivo* so as to further improve the diagnosis and treatment of IBD (Weissleder and Ntziachristos, 2003; Sasaki et al., 2005; Yu et al., 2012; Vegesna et al., 2013; Zhang et al., 2017). A near-infrared (NIR) fluorescent probe has more advantages in non-invasive imaging *in vivo*, which can further enhance the penetration of deep tissue and improve the signal-to-noise ratio (Antaris et al., 2016; Hong et al., 2017; Liu et al., 2021a; Wang et al., 2021; Xu et al., 2021). However, the current small-molecule fluorescent probes used for NO detection still have shortcomings such as short wavelength (<700 nm) and poor water solubility, especially in deep tissue and disease animal models. It is still a great challenge to apply these probes to the real-time imaging detection of NO *in vivo* (Izumi et al., 2009; Li et al., 2016; Zhang et al., 2018).

A near-infrared fluorescent probe (LS-NO) for the real-time detection of NO in inflammatory bowel disease (IBD) was developed by Liu et al. (2021b). The probe used oligoglycol morpholine-functionalized thiophene as strong electron donors and diaminobenzene (1,2,5-thiadiazole) as a weak electron acceptor and NO trapping group. After the specific reaction of the probe with NO, the weak electron acceptor group diaminobenzene (1,2,5-thiadiazole) was transformed into the strong electron acceptor triazolo-benzo-(1,2,5-thiadiazole). By using the enhanced intramolecular charge transfer mechanism, the probe exhibited “off–on”-type near-infrared absorption and emission at 700 and 750/800 nm, respectively. In addition, LS-NO showed good water solubility and optical stability. It can detect exogenous and endogenous NO in the lysosomes of living cells with high sensitivity and specificity. This work suggested that LS-NO was promising as a diagnostic probe for the real-time detection of NO in IBD and may also facilitate inflammatory stool detection. To further understand the fluorescent mechanism and character of the probes LS-NO and LS-TZ (after the reaction of the probe LS-NO with NO), the electron transfer in the excitation and emitting process within the probe model molecules DAD-NO and DAD-TZ was analyzed in detail under the density functional theory. The calculation results indicated the transformation from diaminobenzene (1,2,5-thiadiazole) as the weak electron acceptor to triazolo-benzo-(1,2,5-thiadiazole) as the strong electron acceptor made LS-NO an effective “off–on” NIR NO fluorescent probe.

## 2 Methods

The ORCA 5.0.1 (Neese, 2018) software was used to perform optimization and vibrational frequency analysis on the S_0_ structures of the model probes DAD-NO and DAD-TZ under PBE0/def2-TZVP with D3 dispersion correction (Adamo and Barone, 1999; Weigend and Ahlrichs, 2005; Grimme et al., 2011), and then single-point energy and TDDFT calculation under wB2GP-PLYP/def2-TZVP so as to obtain the free energy with high precision (Goerigk and Grimme, 2014; Casanova-Páez et al., 2019; Casanova-Paez and Goerigk, 2020; Peng et al., 2021; Liu et al., 2022). The functional PBE0-D3(BJ) and wB2GP-PLYP used for structure optimization and TDDFT calculation of such organic probe molecules were verified to be proper (Ali et al., 2020; Bremond et al., 2021; Santra and Martin, 2022). The optimized S_1_ structures of DAD-NO and DAD-TZ were obtained under a combination of wB2GP-PLYP/def2-TZVP to analyze the emitting wavelength in the excitation and radiation process of the probe. All the figures were rendered by means of VMD 1.9.3 software (Humphrey et al., 1995) and the analyses were conducted using the Multiwfn 3.7 code (Lu and Chen, 2012).

## 3 Results and discussion

**FIGURE 1:**
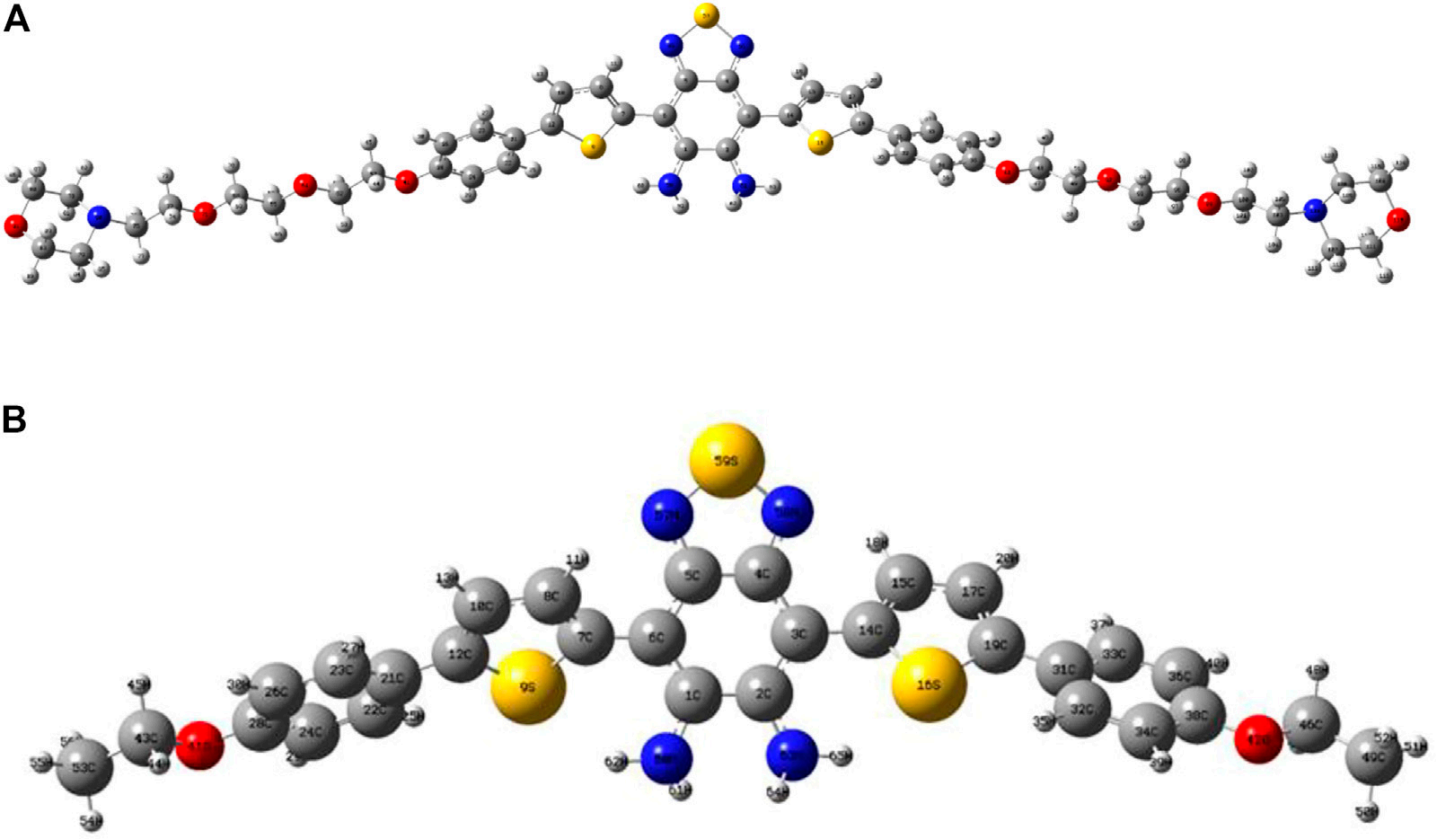
Optimized structures of **(A)** LS-NO and **(B)** DAD-NO.

**FIGURE 2:**
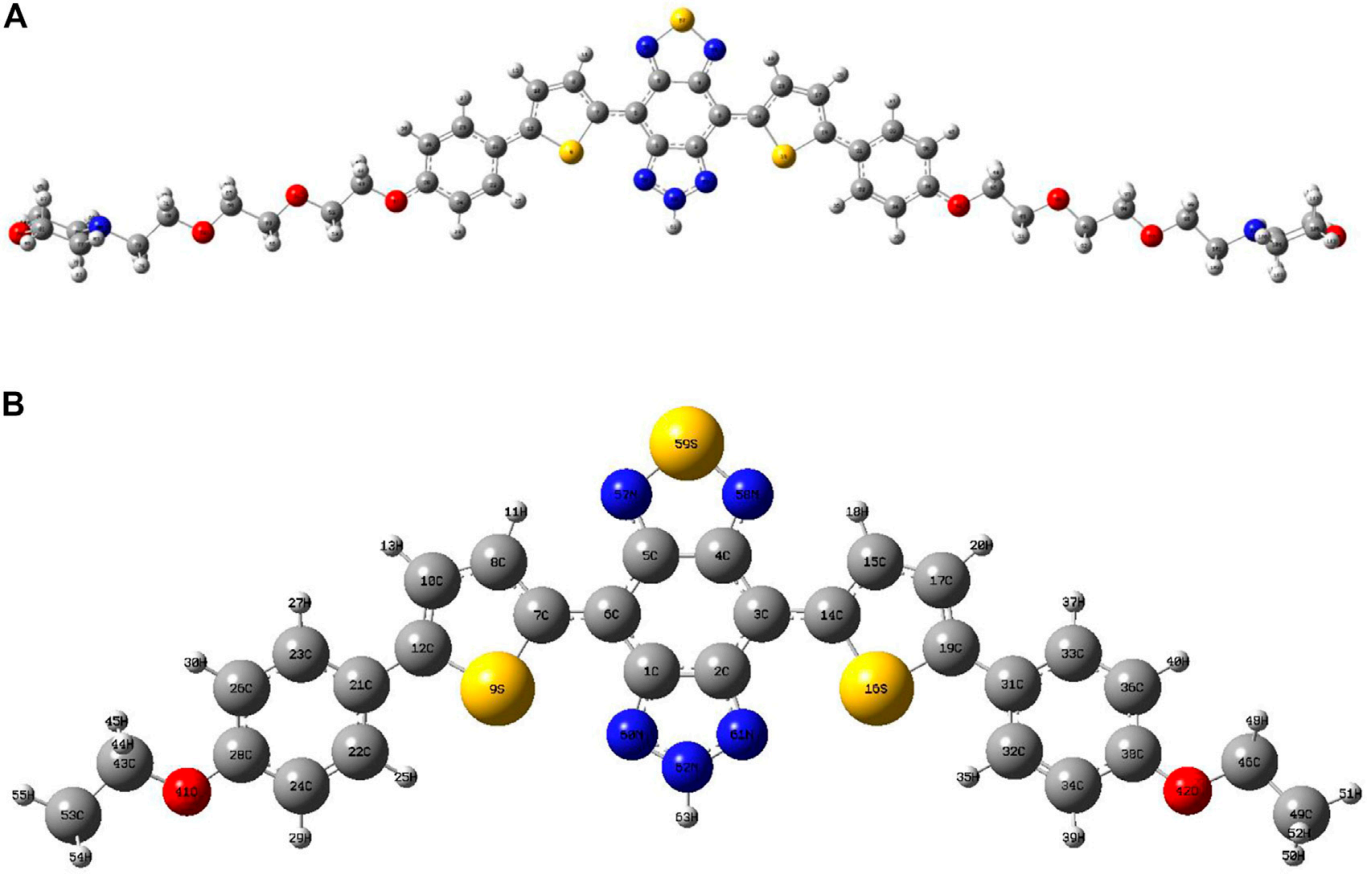
Optimized structures of **(A)** LS-TZ and **(B)** DAD-TZ.

The optimized structures of probes LS-NO and LS-TZ with corresponding model probes DAD-NO and DAD-TZ are depicted in Figures 1, 2, respectively. In order to focus solely on the electron donor and acceptor parts in the probe, and to reduce computational time, the two terminal groups in the probes (LS-NO and LS-TZ) were cut to ethyl as shown in the model probes (DAD-NO and DAD-TZ).

The structures of the probes (as shown in Figures 1, 2) show that the DAD-NO had a more twisted structure than DAD-TZ, while the weak electron acceptor diaminobenzene (1,2,5-thiadiazole) was replaced by triazolo-benzo-(1,2,5-thiadiazole) as a strong electron acceptor.

**FIGURE 3:**
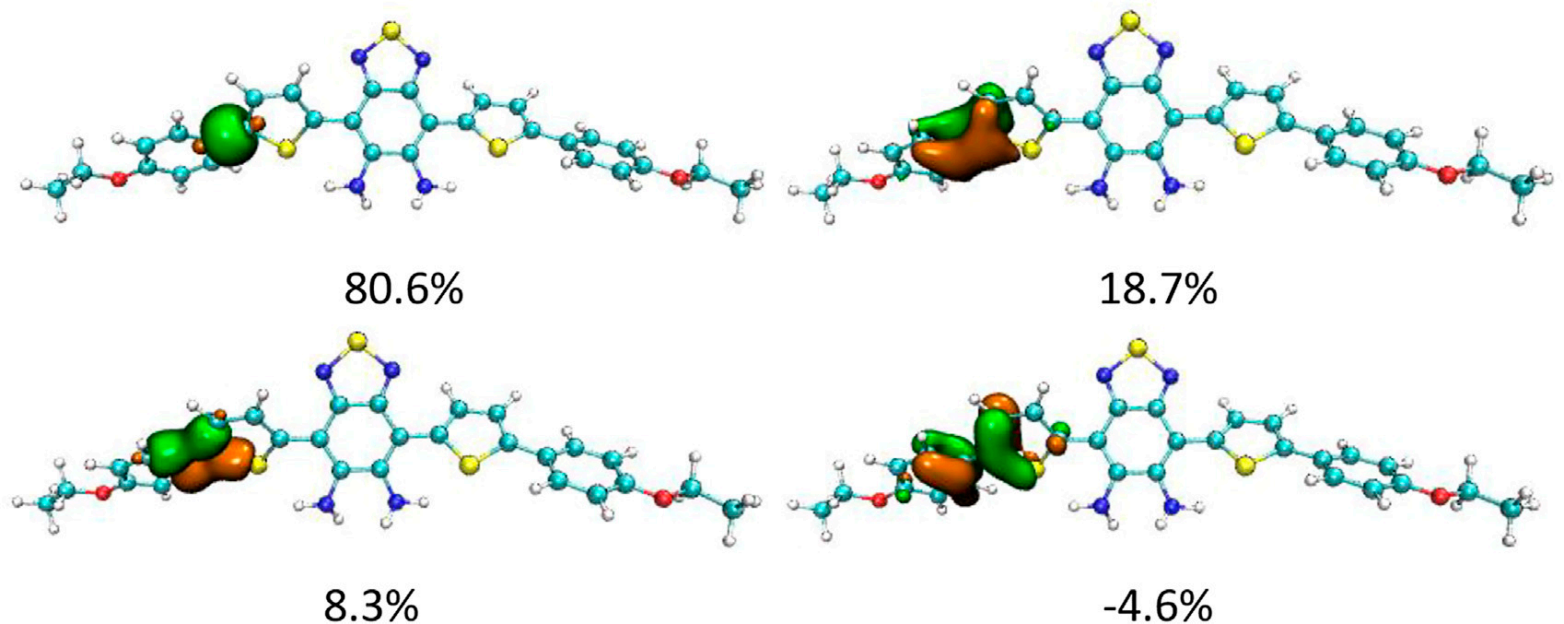
NAdO distribution of the *a*-related C–C bonds in DAD-NO.

**FIGURE 4:**
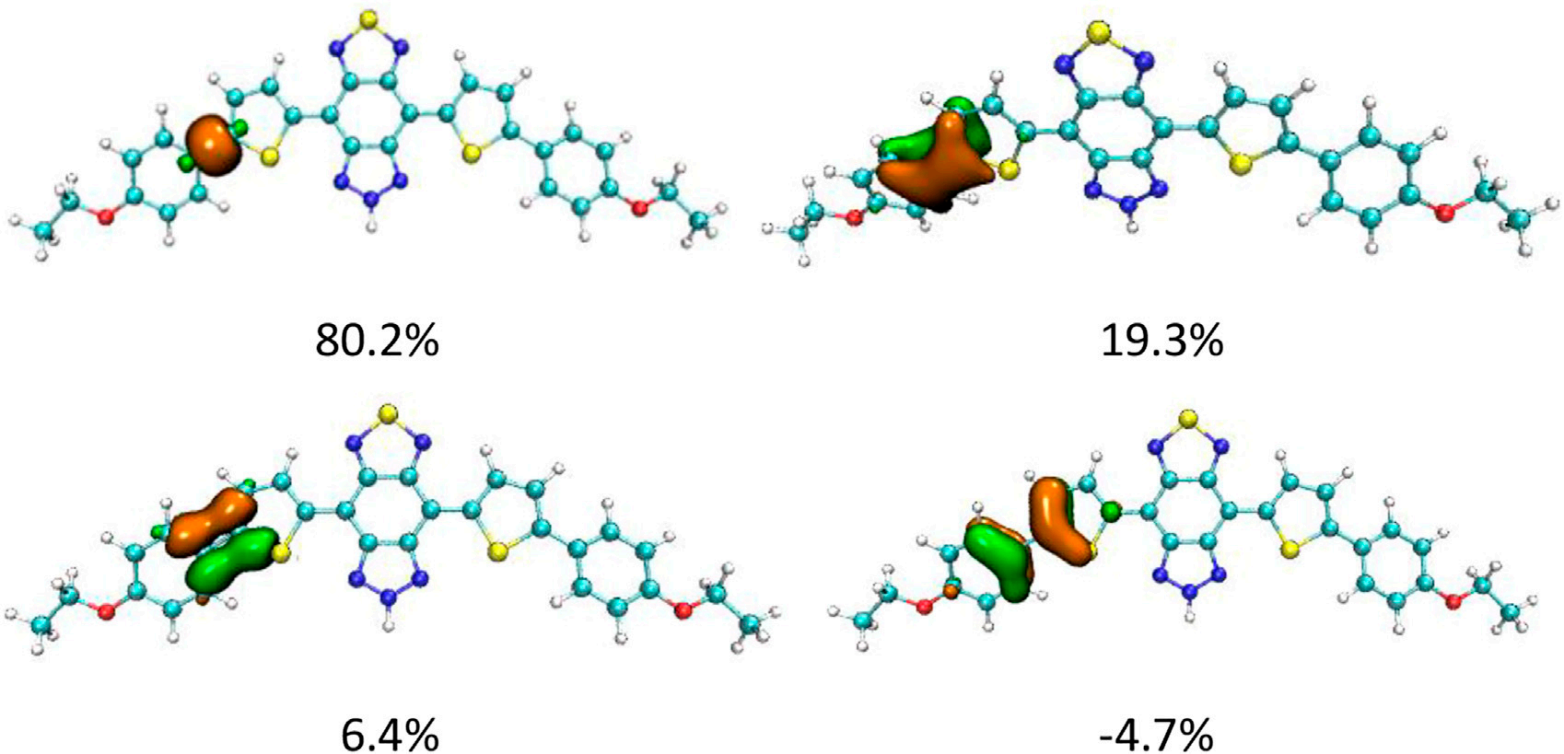
NAdO distribution of the *a*-related C–C bonds in DAD-TZ.

Although DAD-NO had a more twisted structure than DAD-TZ, the α(β)-related C–C bonds shown in Figures 1, 2 were all typical C–C single bonds and had a similar natural adaptive orbital (NAdO) distribution (Zhang et al., 2020). The details of the NAdO distribution of the *a*-related C–C bonds in DAD-NO and DAD-TZ are shown in Figures 3, 4, respectively. The details of the NAdO distribution of *ß*-related C–C bonds in DAD-NO and DAD-TZ are given in Supplementary Figures S1, S2, respectively, for reference.

Figures 3, 4 show that the *a*-related C–C bond was a typical single C–C bond with main contribution from the localized sigma bond—about 80% in both DAD-NO and DAD-TZ. The second large contribution (about 19%) came from the pi bond which delocalized to the neighbor carbon atoms unlike the sigma bond. There were two other NAdOs which consist of *P* orbitals of carbon atoms with parallel and opposite phases, respectively, as shown in Figures 3, 4. The last two NAdOs displayed non-negligible contributions to the C–C bond with positive and negative values, respectively.

**FIGURE 5:**
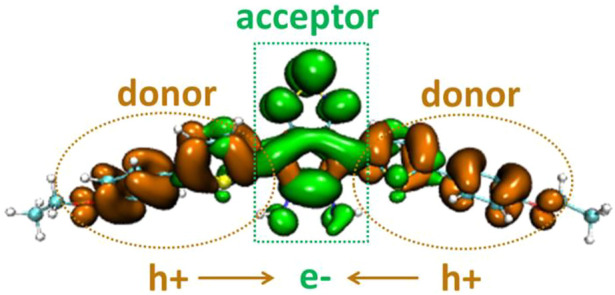
Electron distribution difference between S_0_ and S_1_ of DAD-NO (orange and green in the isosurface map represent the hole and electron distribution in the excitation process).

**FIGURE 6:**
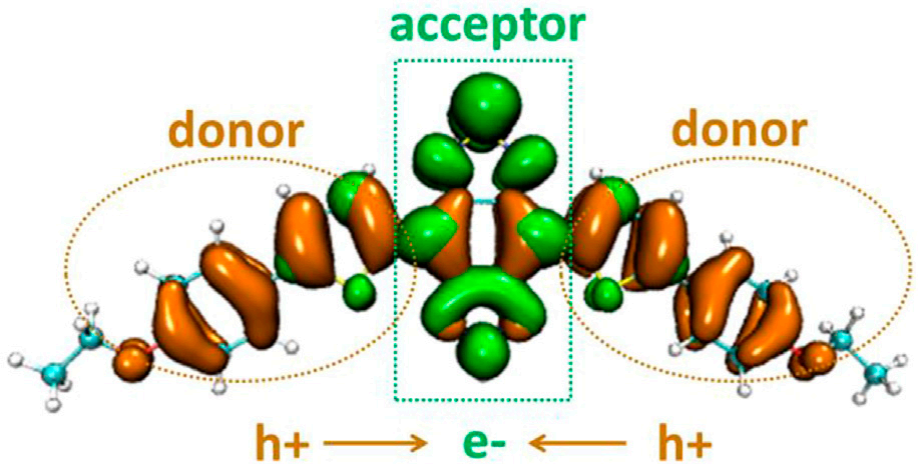
Electron distribution difference between S_0_ and S_1_ of DAD-TZ.

The electron distribution difference between the first excitation state and ground state of DAD-NO and DAD-TZ was obtained using Multiwfn 3.7 and depicted in Figures 5,6, respectively. The electron donor–acceptor–donor character in the probes could be clearly seen from the electron transfer process (from hole “h+" to electron “e−" as shown in Figures 5, 6). The electron acceptor parts in the probes mainly consist of N-contained and central hex-atomic rings, while the oxygen, five-membered and hex-atomic rings on the two sides contributed the donor parts in the probe.

**FIGURE 7:**
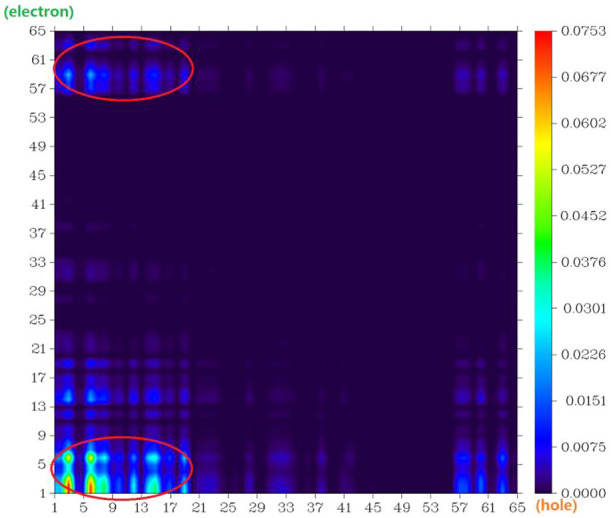
Atom–atom electron transfer heatmap of DAD-NO (atom numbers were referred to Figure 1).

**FIGURE 8:**
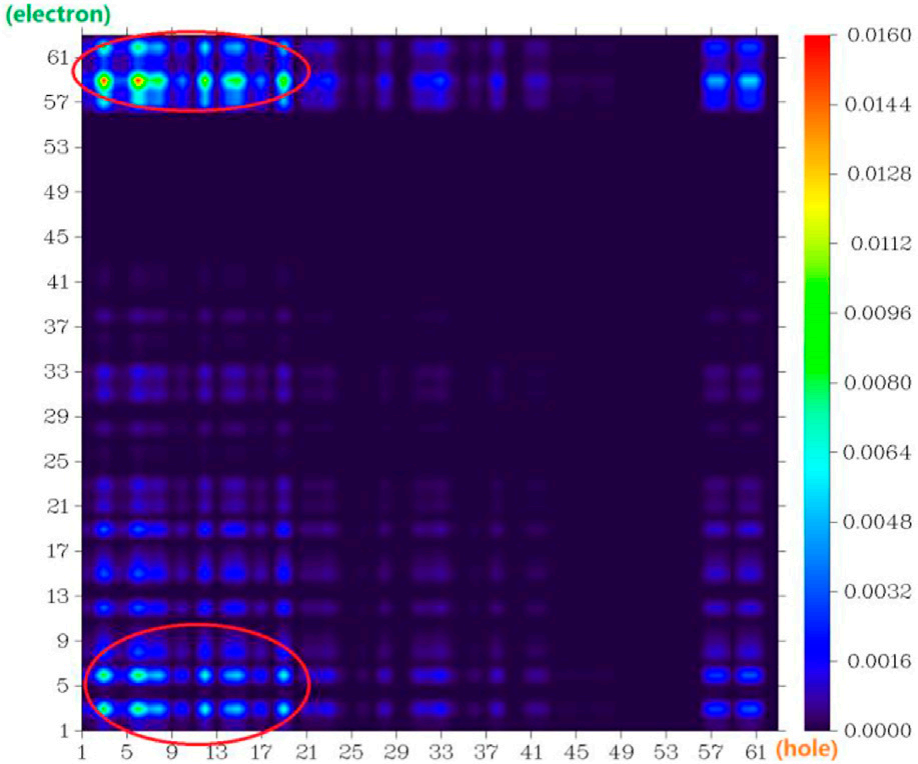
Atom–atom electron transfer heatmap of DAD-TZ (atom numbers were referred to Figure 2).

**FIGURE 9:**
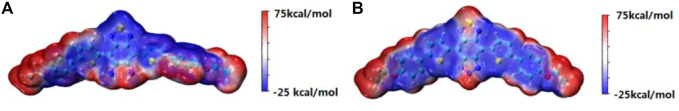
Electrostatic potential of **(A)** DAD-NO and **(B)** DAD-TZ.

The atom–atom electron transfer heatmap in the electron excited process of DAD-NO (Figure 7) and DAD-TZ (Figure 8) clearly indicated the obvious electron transfer from the donor parts to acceptor parts. The electrostatic potential of DAD-NO and DAD-TZ is shown in Figure 9. It could be also seen that the replacement of weak electron acceptor diaminobenzene (1,2,5-thiadiazole) by strong electron acceptor triazolo-benzo-(1,2,5-thiadiazole) made the electron acceptor part of DAD-TZ take a larger electrostatic potential value than DAD-NO.

**FIGURE 10:**
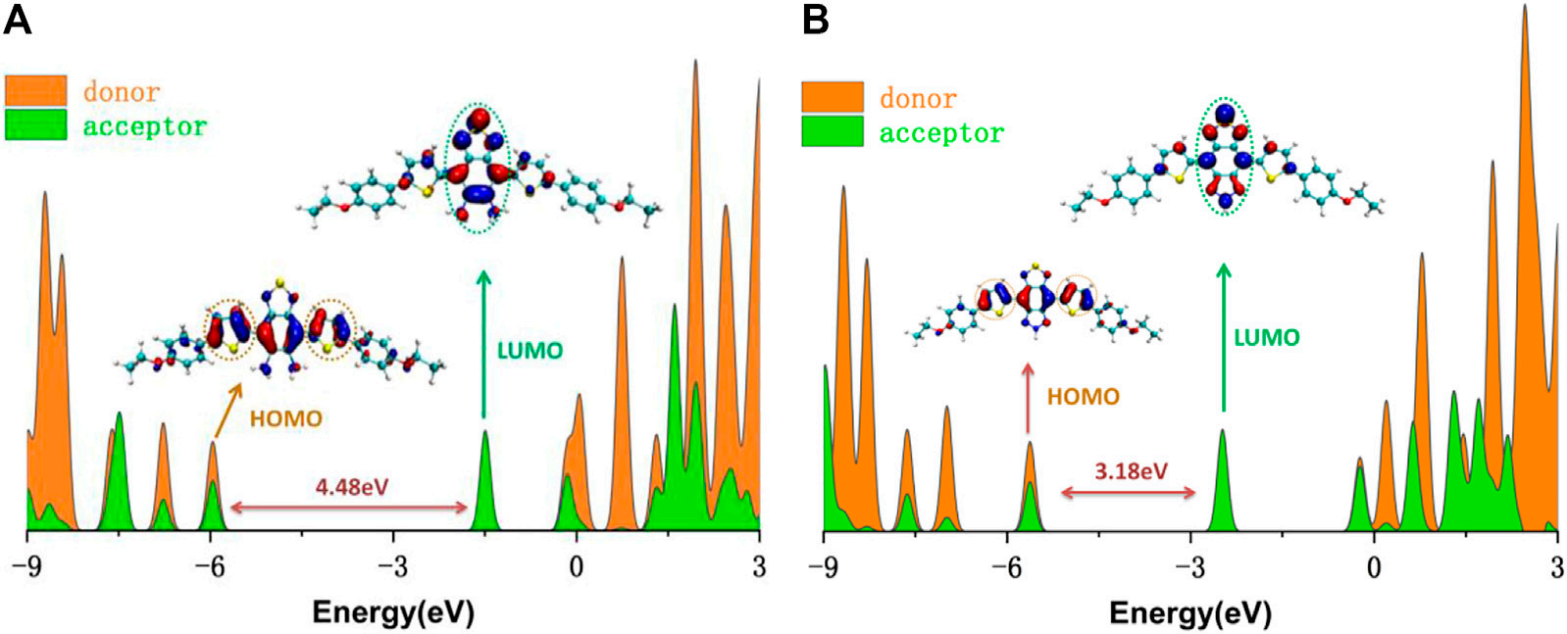
DOS of **(A)** DAD-NO and **(B)** DAD-TZ and the corresponding HOMO-LUMO energy gap calculated at the wB2GP-PLYP/def2-TZVP level in the gas.

To clarify the contribution of the donor and acceptor parts in DAD-NO and DAD-TZ to the density of electronic states, the DOS of DAD-NO and DAD-TZ and the corresponding HOMO-LUMO energy gap calculated at the wB2GP-PLYP/def2-TZVP level in the gas are depicted in Figure 10. It was obvious that the donor part’s contribution to the HOMO exceeded that of the acceptor part, while the opposite situation happened within the LUMO. The replacement of diaminobenzene (1,2,5-thiadiazole) by triazolo-benzo-(1,2,5-thiadiazole) led to a smaller HOMO-LUMO energy gap which made the near-infrared fluorescence generation enhanced in DAD-TZ than in DAD-NO.

**FIGURE 11:**
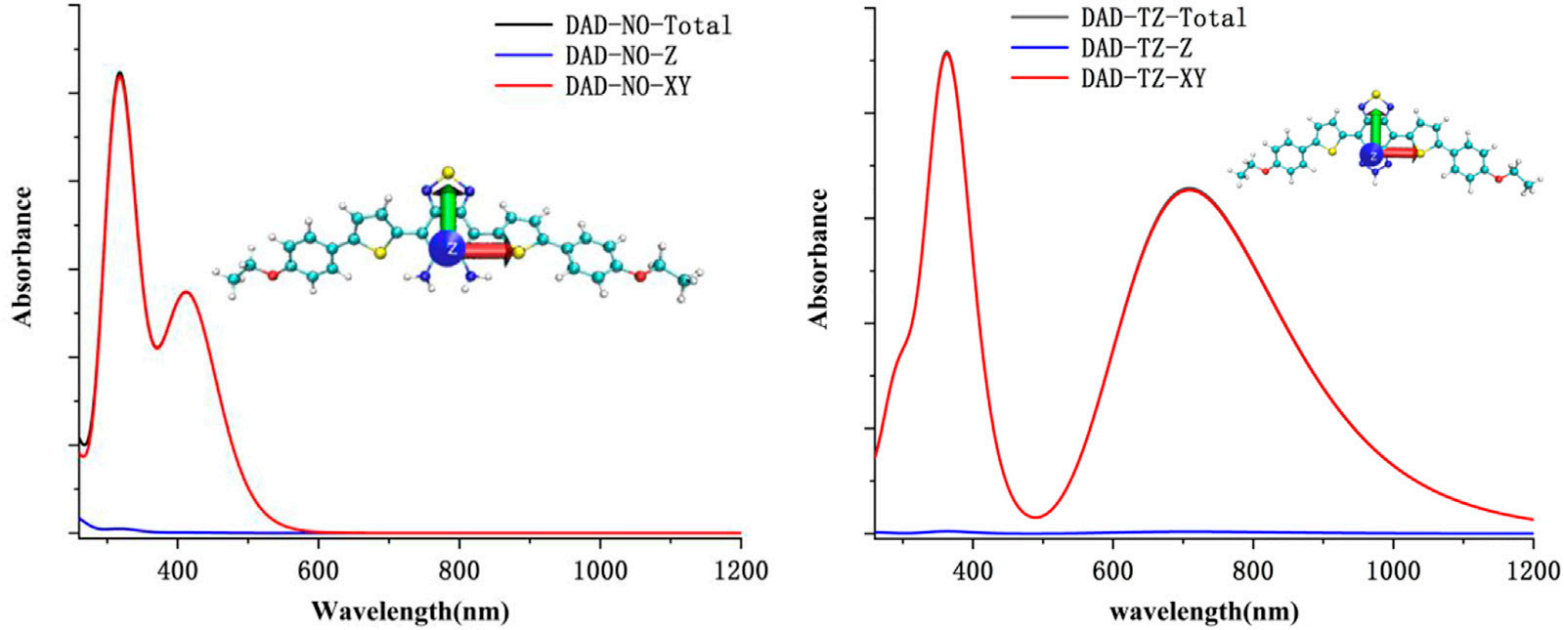
Calculated UV-Vis spectrum of DAD-NO and DAD-TZ in aqueous solution.

**FIGURE 12:**
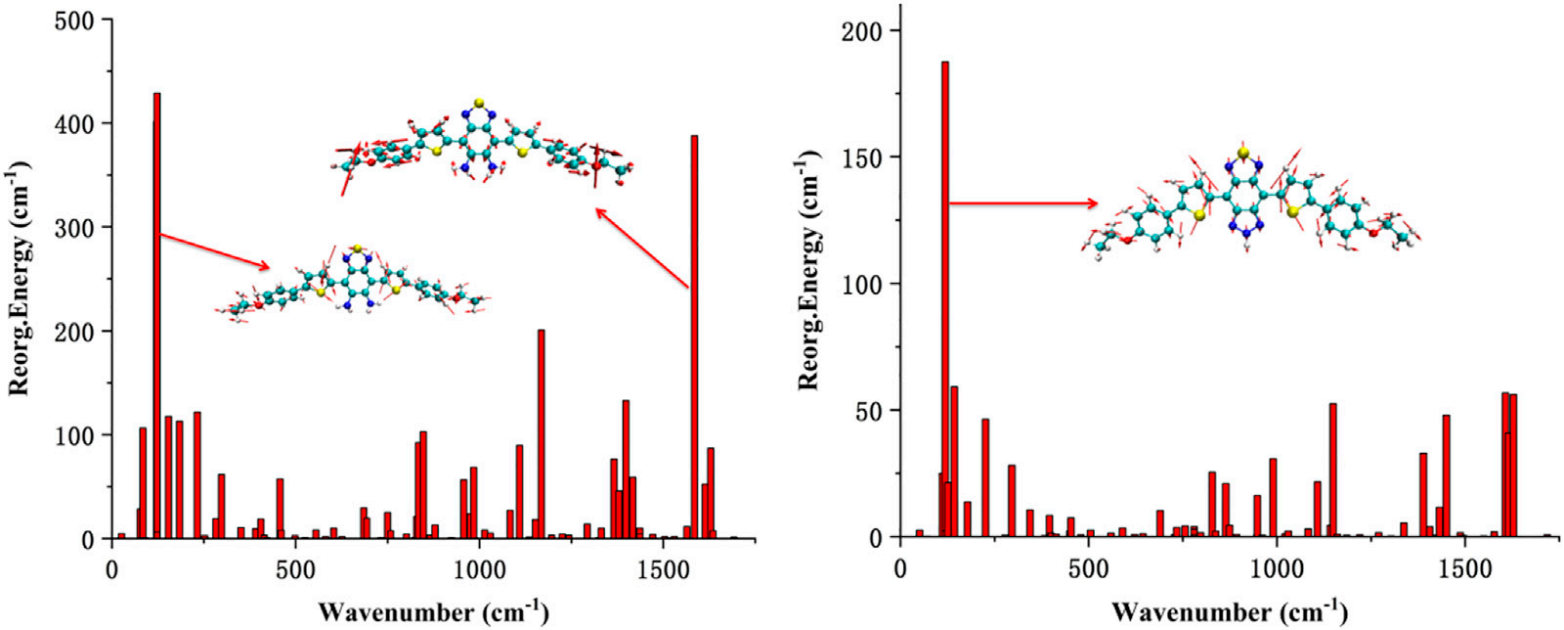
Calculated reorganization energy from each normal mode of DAD-NO and DAD-TZ.

To understand the quantificational change in the energy and spectrum between DAD-NO and DAD-TZ, the UV-Vis spectrum of the probes in aqueous solution (a mixture of DMF/water with a volume ratio of 50/50) was analyzed using TDDFT calculation under the wB2GP-PLYP/def2-TZVP method. The calculated results are shown in Figure 11. As shown in Figure 11, there were absorption peaks located within the red and blue channels for DAD-TZ but only absorption peaks located within blue channels for DAD-NO which was consistent with the experimental results (Liu et al., 2021b). In addition, it could be clearly shown that the energy absorbance mainly located inside the probe molecular plane (XY plane) and the energy absorbance along the perpendicular direction to the molecular plane (*Z* axis) were almost negligible. This conclusion was consistent with the reorganization energy analysis between the ground and first excited states of DAD-NO and DAD-TZ in Figure 12. It could be clearly shown that the reorganization energy of DAD-NO was bigger than that of DAD-TZ, while the direction of the norm modes with most contribution were both parallel to the molecular plane in the two probes.

## 4 Conclusion

The geometric and electronic structures of the ground and excited states of an effective “off–on” NIR NO fluorescent model probe DAD-NO (DAD-TZ) were analyzed under the density functional theory in detail. The calculated results indicated that the transformation from diaminobenzene (1,2,5-thiadiazole) as a weak electron acceptor in DAD-NO to triazolo-benzo-(1,2,5-thiadiazole) as a strong electron acceptor in DAD-TZ made DAD-NO an effective “off–on” NIR NO fluorescent probe with high sensitivity and specificity. The electrostatic potential and density of electronic state analysis also suggested the changing of the electron acceptor part within DAD-NO, and DAD-TZ was the structural origin of the switch on/off of the NIR fluorescence in the probes. Energy absorbance mainly located inside the probe molecular plane (XY plane) and energy absorbance along the perpendicular direction to the molecular plane (*Z* axis) were almost negligible. All these theoretical results would provide an insight for designing new effective probes with similar functions.

## Data Availability

The original contributions presented in the study are included in the article/Supplementary Material; further inquiries can be directed to the corresponding author.
