# Supplementary material for: Thiophene and diaminobenzo- (1,2,5-thiadiazol)- based DAD-type near-infrared fluorescent probe for nitric oxide: A theoretical research
Source: Front Chem. 2023 Jan 9;10:990979. doi: 10.3389/fchem.2022.990979 (PMC9870051; doi:10.3389/fchem.2022.990979)
Supplement: Supplementary file 1 [file DataSheet1.docx]

Density Functional Theory Studies on Thiophene and Diaminobenzo-(1,2,5-thiadiazol)-based D-A-D type Near-Infrared Fluorescent Probe for Nitric Oxide

XY Lin^1^, SH Sun^1^, YT Liu^1^, QQ Shi^1^, JJ Lv^1^, YJ Peng*^2^

1. College of Public Health, Jinzhou Medical University, Jinzhou 121001, P. R. China
2. College of Bio-informational engineering, Jinzhou Medical University, Jinzhou 121001, P. R. China


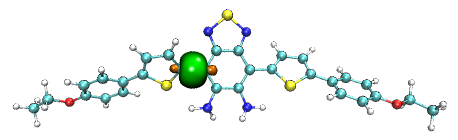

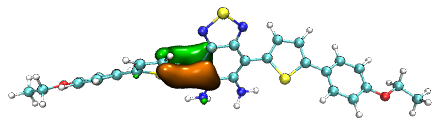


81.1% 18.2%


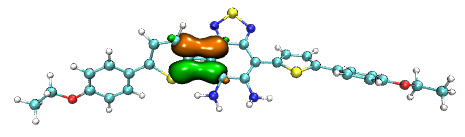

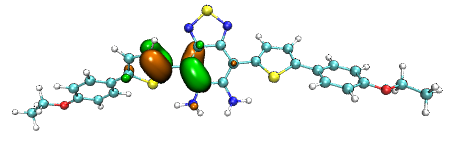


6.7% -4.6%

Figure S1 NAdO orbital distribution in the β-related C-C bonds in DAD-NO


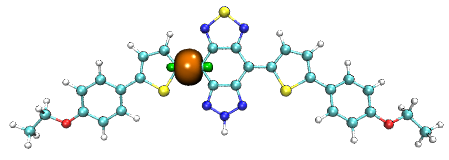

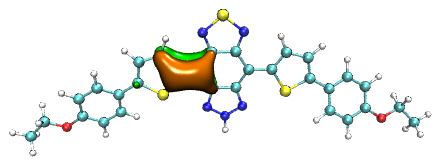


77.3% 23.6%


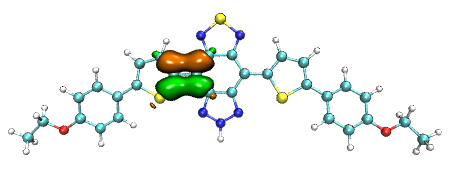

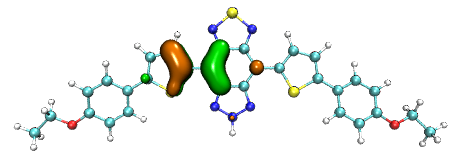


6.1% -5.6%

Figure S2 NAdO orbital distribution in the β-related C-C bonds in DAD-TZ

Original input files of DAD-NO and DAD-TZ:

! PBE0 opt freq d3 def2-TZVP def2/J def2-TZVP/C RIJCOSX grid6 gridx6 tightSCF noautostart miniprint nopop

* xyz 0 1

C -1.26702200 -2.22827300 0.09140300

C 0.17945600 -2.22877100 -0.07569000

C 0.91250600 -1.05377500 -0.18209200

C 0.17202700 0.17266700 -0.11586900

C -1.26168600 0.17346100 0.10093600

C -2.00118600 -1.05271500 0.18234300

C -3.45596100 -1.00661700 0.31389400

C -4.21257300 -0.11977200 1.04804500

S -4.50992600 -2.06551100 -0.59045300

C -5.60869900 -0.30782700 0.90504500

H -3.75833700 0.66010100 1.65692800

C -5.94651100 -1.33376300 0.05023300

H -6.35334600 0.31573300 1.40101900

C 2.36710900 -1.00815400 -0.31615400

C 3.12171800 -0.13123000 -1.06413200

S 3.42346000 -2.05381700 0.60068900

C 4.51826800 -0.31664300 -0.92150900

H 2.66577200 0.63977400 -1.68295700

C 4.85831400 -1.33042300 -0.05322600

H 5.26168600 0.30020300 -1.42763700

C -7.27068900 -1.82005600 -0.33064800

C -7.48045000 -2.54252800 -1.51995400

C -8.38550500 -1.57653000 0.48198700

C -8.73870200 -2.99623100 -1.87584100

H -6.63721900 -2.73638200 -2.18830300

C -9.65831200 -2.01850500 0.13144200

H -8.25662000 -1.04240800 1.42568100

C -9.84598700 -2.73685900 -1.05561800

H -8.89964500 -3.55219900 -2.80122400

H -10.49450200 -1.80789700 0.79819100

C 6.18354900 -1.81126300 0.33092400

C 6.39746100 -2.51276200 1.53197200

C 7.29511500 -1.58359600 -0.49068400

C 7.65670500 -2.96168400 1.89046500

H 5.55680600 -2.69356800 2.20718500

C 8.56887700 -2.02098000 -0.13782800

H 7.16264900 -1.06628400 -1.44321500

C 8.76075400 -2.71843200 1.06097100

H 7.82097700 -3.50133800 2.82487800

H 9.40242600 -1.82344600 -0.81184700

O -11.03122700 -3.21150700 -1.48219600

O 9.94721700 -3.18715700 1.49069200

C -12.18622600 -2.97505000 -0.71093500

H -12.06208300 -3.41000500 0.29874100

H -12.33843900 -1.88675400 -0.58284800

C 11.09894200 -2.96680700 0.70983700

H 10.96920000 -3.41968700 -0.29121800

H 11.25250000 -1.88129200 0.56137900

C 12.27522500 -3.58108100 1.42652100

H 12.12265300 -4.66121800 1.56539200

H 13.19729400 -3.43047300 0.84666400

H 12.40701100 -3.12263200 2.41732200

C -13.36022900 -3.60007800 -1.42204600

H -13.20906000 -4.68283400 -1.54056600

H -14.28479000 -3.43728700 -0.84949900

H -13.48635800 -3.15944400 -2.42162900

N -1.77517700 1.39566900 0.17490200

N 0.68449800 1.39427800 -0.20530900

S -0.54574100 2.44951700 -0.02185600

N -1.83021200 -3.48339100 0.20515800

H -1.46823800 -4.16608600 -0.45395200

H -2.83887400 -3.51582100 0.29767300

N 0.74370100 -3.48462400 -0.17334600

H 0.38098600 -4.15974000 0.49306700

H 1.75243300 -3.51765500 -0.26466400

*

! PBE0 opt freq d3 def2-TZVP def2/J def2-TZVP/C RIJCOSX grid6 gridx6 tightSCF noautostart miniprint nopop

* xyz 0 1

C -1.25702700 -1.57533200 -0.05965500

C 0.17778700 -1.57409100 0.05346300

C 0.97744500 -0.41469200 0.12072700

C 0.19282800 0.77687300 0.06293700

C -1.27743400 0.77559800 -0.05264300

C -2.05932800 -0.41731900 -0.11882500

C -3.49279500 -0.43841600 -0.23162700

C -4.36759400 0.64127300 -0.30748000

S -4.39162400 -1.93407400 -0.28744000

C -5.71639400 0.26267000 -0.41334200

H -4.00915700 1.66817200 -0.29261800

C -5.90604900 -1.10902100 -0.42171700

H -6.53430700 0.97755400 -0.50762600

C 2.41096700 -0.43330400 0.23327600

C 3.28329500 0.64782300 0.31671500

S 3.31323400 -1.92725500 0.27832900

C 4.63297600 0.27157200 0.41970700

H 2.92249900 1.67397500 0.30920400

C 4.82578000 -1.09970300 0.41827200

H 5.44926200 0.98764100 0.51898000

C -7.15782200 -1.85146900 -0.51888900

C -7.18570100 -3.21158100 -0.88444000

C -8.38649400 -1.23152700 -0.25086500

C -8.37631000 -3.90967200 -0.97702800

H -6.24934200 -3.72616900 -1.11531900

C -9.59199300 -1.92004200 -0.34631300

H -8.40539000 -0.18496300 0.05955600

C -9.59710300 -3.27208200 -0.71128600

H -8.39494300 -4.96228400 -1.26507400

H -10.52101400 -1.39537500 -0.12332900

C 6.07927000 -1.83995000 0.50994000

C 6.11034700 -3.20254500 0.86585600

C 7.30645700 -1.21531600 0.24603500

C 7.30257100 -3.89854900 0.95323000

H 5.17522200 -3.72089000 1.09330600

C 8.51354900 -1.90172900 0.33633900

H 7.32288300 -0.16654300 -0.05698500

C 8.52183900 -3.25630500 0.69172600

H 7.32367900 -4.95312600 1.23381900

H 9.44131400 -1.37337400 0.11686000

O -10.70453900 -4.02713700 -0.82894600

O 9.63102600 -4.00964000 0.80377500

C -11.96475100 -3.44665500 -0.58319600

H -12.00431800 -3.05358300 0.45013500

H -12.12587000 -2.59142200 -1.26617200

C 10.88985200 -3.42452500 0.56194400

H 10.92831400 -3.02398900 -0.46855900

H 11.04914300 -2.57382700 1.25098500

C 11.94484500 -4.48303400 0.76453800

H 11.78787600 -5.32319000 0.07263600

H 12.94557600 -4.06457500 0.58416400

H 11.90944400 -4.87299300 1.79210800

C -13.01727200 -4.50610800 -0.79356300

H -12.85847900 -5.35084100 -0.10767700

H -14.01899100 -4.09125700 -0.61036800

H -12.98081000 -4.88860500 -1.82389700

N -1.77748300 2.01781500 -0.08738300

N 0.69003700 2.01995400 0.10639100

S -0.54490200 3.05303500 0.01312800

N -1.65724700 -2.86579000 -0.09566300

N 0.58095200 -2.86385200 0.08044300

N -0.53739500 -3.52449300 -0.00992000

H -0.53623600 -4.54042700 -0.01346700

*
